# Se méfier des lentilles cosmétiques

**DOI:** 10.11604/pamj.2014.17.143.4095

**Published:** 2014-02-28

**Authors:** Hakima Elouarradi, Lalla Ouafae Cherkaoui

**Affiliations:** 1 Université Mohammed V Souissi, service d'Ophtalmologie A de l'hôpital des Spécialités, Centre hospitalier universitaire, Rabat, Maroc

**Keywords:** Lentilles de contact, infection, abcès de cornée, Contact lenses, infection, corneal abscess

## Image en medicine

Les lentilles de contact cosmétiques comme celles à visée optique exposent aux complications infectieuses dont la plus redoutable est l'abcès de cornée qui met en jeu le pronostic visuel. Leur vente bénéficie toutefois d'un statut particulier vis-à-vis de la législation dans la mesure où elles sont assimilées à des produits cosmétiques et non à des dispositifs médicaux. Les kératites infectieuses chez les porteurs de lentilles cosmétiques ne sont pas rares et peuvent avoir des conséquences dramatiques. Elles sont causées par divers agents bactériens, parasitaires ou plus rarement viraux et mycosiques. Le manque d'hygiène et d'entretien des lentilles de contact apparait comme un des principaux facteurs de risque. Nous présentons 2 cas de kératites infectieuses survenues chez des porteuses de lentilles cosmétiques. Les patientes sont âgées de 16 et 30 ans. Le prélèvement bactériologique est positif avec pseudomonas aeruginosa dans les 2 cas. Patientes mises sous collyres fortifiées d'antibiotiques horaires (vancomycine et ceftazidime) avec évolution favorable au prix d'opacité cornéenne cicatricielle dans les 2 cas. Une corticothérapie locale est instaurée secondairement pour réduire l'opacité et la néo vascularisation cornéenne avec surveillance régulière et stricte. La prévention des kératites infectieuses chez les porteurs de lentilles cosmétiques passe par une information éclairée des patients sur la manipulation et l'entretien de leurs lentilles et la modification de la législation sur la vente des lentilles cosmétiques qui n'est soumise à aucun contrôle comme l'ont déjà fait plusieurs pays devant le risque de survenue d'incidents graves.

**Figure 1:**
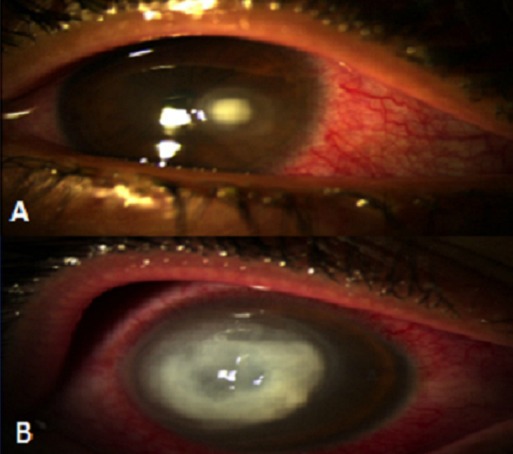
A) Abcès para central bien limité, avec œdème péri lésionnel; B) Abcès cornéen avec œdème périlésionnel et descemétocèle centrale

